# Predictive Role of the p16 Immunostaining Pattern in Atypical Cervical Biopsies with Less Common High Risk HPV Genotypes

**DOI:** 10.3390/diagnostics11111947

**Published:** 2021-10-20

**Authors:** Daniela Cabibi, Caterina Napolitano, Antonino Giulio Giannone, Maria Carmela Micciulla, Rossana Porcasi, Roberta Lo Coco, Liana Bosco, Manlio Vinciguerra, Giuseppina Capra

**Affiliations:** 1 Anatomic Pathology Unit, Department of Health Promotion, Mother and Child Care, Internal Medicine and Medical Specialties (PROMISE), University of Palermo, 90127 Palermo, Italy; cabibidaniela@virgilio.it (D.C.); catera.napolitano@gmail.com (C.N.); mariacarmelamicciulla@gmail.com (M.C.M.); r.porcasi@libero.it (R.P.); lococo.roberta@gmail.com (R.L.C.); giuseppina.capra@unipa.it (G.C.); 2 Section of Biology and Genetics, Department of Biomedicine, Neuroscience and Advanced Diagnostics (Bi.N.D), University of Palermo, 90127 Palermo, Italy; liana.bosco@unipa.it; 3 Department of Biological, Chemistry and Pharmaceutical Sciences and Technologies (STEBICEF), University of Palermo, 90127 Palermo, Italy; 4 International Clinical Research Center, St. Anne’s University Hospital, 656 91 Brno, Czech Republic; manliovinciguerra@gmail.com; 5 Division of Medicine, Institute for Liver and Digestive Health, University College London (UCL), London WC1E 6BT, UK; 6 Department of Translational Stem Cell Biology, Research Institute of the Medical University of Varna, 9002 Varna, Bulgaria

**Keywords:** high-risk HPV, p16, Ki67, immunohistochemistry

## Abstract

P16 immunostaining is considered a useful surrogate of transcriptionally active high-risk (hr) HPV infection. Only strong and widespread “block-like” immunoreactivity is considered specific, whereas weak/focal p16 positive immunostaining is considered not specific, and follow-up and HPV molecular detection is not indicated. The aim of the study was to evaluate the presence of HPV DNA and Ki67 immunostaining in 40 cervical atypical biopsies (CALs) with mild and focal histological features suggestive of HPV infection—20 cases with weak/focal p16 positive immunoreactivity and 20 cases negative for p16 expression. In 16/20 weak/focal p16 positive CALs (80%), the INNO-LiPA HPV genotyping detected hrHPV genotypes (HPV 31, 51, 56, 59, 26, 53, 66, 73, and 82). Co-infection of two or more hrHPV genotypes was often evidenced. HPV16 and 18 genotypes were never detected. Ki67 immunostaining was increased in 10/20 cases (50%). In 19/20 p16 negative CALs, hrHPV infection was absent and Ki67 was not increased. These results suggest that weak/focal p16 immunostaining represents the early stage of transcriptionally active infection, strongly related to the presence of less common hrHPV genotypes, probably with a slower transforming power, but with a potential risk of progression if the infection persists. HPV DNA genotyping and follow-up could be useful in these cases to verify if they are able to evolve into overt dysplastic changes and to improve knowledge of less common hrHPV genotypes.

## 1. Introduction

The etiological role of HPV infection in cervical cancer is well known. To date, more than 200 HPV types have been completely sequenced, characterized, and numbered. Among these, approximately 40 can infect the human mucosa, above all the anogenital and aerodigestive tracts [1].

The International Agency for Research on Cancer (IARC) classified 12 HPV types as human carcinogens (Group 1), which are commonly referred to as “high risk-HPVs” [2] and represent the main (virtually 100%) etiological agents of this neoplasia [3].

High-risk (hr) HPV types include HPV 16, 18, 31, 33, 35, 39, 45, 51, 52, 56, 58, and 59; probable or possible hrHPV types (IARC Group 2A and 2B) include HPV 26, 53, 66, 67, 68, 70, 73, and 82; and low-risk (lr) HPV types include HPV 6, 11 (IARC Group 3), 40, 42, 54, 55, 61, 62, 64, 69, 71, 72, 81, 83, 84, and 89 [1,4]

Many women get cervical HPV infections, and most do not progress to cervical cancer because infections are transient and viral clearance by the immune system occurs spontaneously without ever causing lesions. High risk HPV infections that “persist” are more likely to evolve into high-grade squamous intraepithelial lesion (HSIL). The evolution to cervical carcinoma may take several years if left untreated [5].

The integration of HPV into the cell genome is considered one of the main factors for HSIL and cervical carcinoma development [6,7].

The pathogenesis of HPV infection consists in an overexpression of E6 and E7 oncoproteins, which can interact with a variety of cellular proteins, inducing proliferation and, eventually, malignant transformation.

The best-regarded interactions are with TP53 and pRB, both master regulators of the cell cycle and mutated in many human cancers.

Degradation of p53 by E6 oncoprotein allows cells to continue replicating [8]. Binding of E7 causes disruption and loss of RB/E2F repressor complex, releasing E2F- transcription factor. This leads to the overexpression of the Cyclin-Dependent kinase inhibitor 2A (CDKN2A/p16) gene, which allows entry into the S phase of the cell cycle [9].

Up-regulation of CDKN2A leads to p16 expression, absent in the normal cervix, as the host’s response to deactivated pRB by HPV proteins. So, the immunohistochemical assessment of p16 is considered a surrogate for transcriptionally active hrHPV infection.

The expression of this marker in the cervical epithelium is of diagnostic value only when a diffuse and intense positivity is observed, so called “block-like positivity” [10,11,12].

Weak and focal p16 positive (w/f p16+) immunostaining is considered not specific, because it has also been reported in reactive squamous epithelium [12].

Ki67 is a nuclear antigen associated with cell proliferation. The protein is present during all active phases of the cell cycle, but is absent in resting cells (G0). In the normal exocervical epithelium, Ki67 is localized within the basal and suprabasal layers, whereas in squamous intraepithelial lesions it is increased [11,13]. So, block-like p16 expression and Ki67 increase are considered very useful for the initial assessment of cervical biopsies with histologically indeterminate dysplasia [11], to submit them to molecular HPV DNA detection (HPV DNA test).

In cervical biopsies lacking obvious dysplastic alterations, w/f p16+ immunostaining does not impose either HPV DNA testing or strict follow-up.

Previous studies have demonstrated that, despite the use of strict histologic criteria for the diagnosis of CIN 1 lesions, there was a false positive rate, or an overinterpretation of CIN 1, in up to 34% of cervical atypical biopsies that, via molecular methods, were negative for HPV infection [14].

In cervical biopsies lacking obvious dysplasia, defined as “cervical atypical lesions” (CALs), a significant statistical correlation between the Ki67 expression in the higher layers of cervical squamous epithelium and the presence of hrHPV was reported [15].

As some CALs show w/f p16+ immunostaining, the aim of this study is to correlate w/f p16+ immunostaining with Ki67 immunoreactivity and to perform HPV DNA testing to investigate the presence of HPV DNA so as to better understand the real meaning of w/f p16+ in cervical biopsies.

## 2. Materials and Methods

The study was carried out on 20 formalin fixed, paraffin embedded cervical biopsies with histological features of CALs showing weak and focal p16 immunostaining (w/f p16+ CALs), and 20 CALs negative for p16 immunostaining (p16- CALs), consecutively received between June and December 2020. Patients’ ages ranged from 27 to 53 (mean age 35).

Histological features of HPV infection (namely, koilocytosis, multinucleation, acanthosis, papillomatosis, parakeratosis, individual cell keratinization, mitoses in the lower basal third of the epithelium, hyperplasia of basal layers, and the absence of distinct basal cell layer) were inconsistently present or only weakly/focally evident, raising the question of a differential diagnosis between HPV-associated changes and repair/reactive or metaplastic changes. Cases with obvious dysplasia and/or with “block-like” p16 expression were excluded.

A “block-like” p16 pattern was considered when strong/diffuse nuclear and cytoplasmic immunoreactivity represented greater than or equal to 50% reactivity in all the epithelium layers. A weak and focal p16 expression was considered when weak/focal/scattered nuclear and cytoplasmic positivity was present in more than 10 basal/parabasal cells and in less than 50% of the epithelium thickness.

Immunohistochemical stains were carried out with a BenchMark ULTRA automated slide staining system (Ventana Medical Systems, Tucson, AZ, USA) according to the manufacturer’s instructions, using the following primary antibodies: p16 (clone E6H4, Ventana Medical Systems, Tucson, AZ, USA) and Ki67 (clone 30-9, Ventana Medical Systems, Tucson, AZ, USA). For all the immunohistochemical stains, the 3,3-diaminobenzidine kit was used as chromogen. The slides were observed on a Leica DM2000 microscope (Leica Microsystems, Wetzlar, Germany). The microphotographs were obtained using a Leica DFC320 camera (Leica Microsystems, Wetzlar, Germany).

For the HPV detection and genotyping of each biopsy, two or three 5 µm paraffin-embedded sections were placed in a microcentrifuge tube with 200 μL of Feoli-Fonseca buffer (0.5% Tween 20, Tris -HCl 50 mM pH 8.5, and 1 mM ethylen diaminetetraacetic-acid) containing 300 μg/mL of proteinase K. The mixture was incubated at 56 °C overnight. Proteinase K was then denatured at 95 °C for 10 min, and after centrifugation at 13,000 rpm for 5 min the supernatant was purified with QIAmp DNA mini kit (QIAgen), following protocol for DNA purification from blood or body fluids.

The DNA quality was checked by β-globin amplification. The INNO-LiPA HPV Genotyping Extra II kit (Fujirebio Diagnostics, Inc, Great Valley Parkway, PA, USA, Malven) was used, based on the combined use of PCR SPF10 and LiPA hybridization. SPF general primers detected at least 43 different HPV genotypes [15]. The LiPA test identified 32 types. There were 20 high risk HPVs found (hrHPV), namely, HPV16, HPV18, HPV26, HPV31, HPV33, HPV35, HPV39, HPV45, HPV51, HPV52, HPV53, HPV56, HPV58, HPV59, HPV68, HPV66, HPV67, HPV70, HPV73, and HPV82, and 12 low risk HPV (lrHPV), namely, HPV6, HPV11, HPV40, HPV42, HPV43, HPV44, HPV54, HPV61, HPV62 HPV81, HPV83, and HPV89 [16,17].

As negative controls, 10 p16 negative surgical cervical samples of uteri resected for the presence of leiomyoma were assessed with an HPV DNA test and Ki67 immunostaining. They appeared histologically normal.

Statistical analysis was performed by using a Chi-square test with Yates correction. Statistical significance was assumed for *p* values < 0.05.

## 3. Results

### 3.1. w/f p16+ CALs

HPV DNA test: 16/20 w/f p16+ CALs (80%) were positive for at least one genotype among hrHPV or probable hrHPV, one case (5%) was positive for lrHPV, and in three cases (15%) no HPV DNA was detected. Ten different HPV genotypes were identified: four hrHPV (genotypes 31, 51, 56, and 59), five probable hrHPV (genotypes 26, 53, 66, 73, and 82) for Groups 1 and 2 of IARC classification, respectively, and only one lrHPV (genotype 54).

In 11/20 cases (55%), at least one of the IARC Group 1 subtypes was identified. The most frequent HPV subtypes were 51, 31, and 73 (each one found in 4/20 cases (20%)). Multiple HPV genotype infections were found in 7/20 (35%) cases. The subtypes most often involved in co-infections were 51 (four cases), 66, 31, and 73 (three cases for each); HPV 51 and HPV 66 were present only in co-infection cases and never alone. Of note, the HPV16 and 18 genotypes were never found (Table 1).

Ki67 immunostaining: 10/20 (50%) w/f p16+ CALs showed Ki67 nuclear positivity limited to the basal/parabasal layer of the squamous epithelium.

In addition, 10/20 cases (50%) showed nuclear positivity for Ki67 in all layers of the squamous epithelium.

Among the latter, 9/10 (90%) showed hrHPV (or probable hrHPV) genotypes.

**Figure 1:**
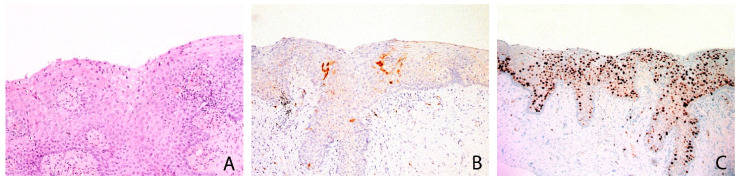
w/f p16+ CAL case showing koilocytic changes and mild epithelial atypia (**A**) with a focal positivity for p16 in few epithelial cells (**B**) and high proliferation rate with Ki67 positive immunostaining in all the squamous epithelium (**C**). A: Ematoxylin and eosin, 200×; B: p16 immunohistochemical stain, 100×; C: Ki67 immunohistochemical stain, 100×.

Among all hrHPV positive cases, 9/16 (56.3%) showed a high proliferation rate with Ki67 positive immunostaining in all of the squamous epithelium layers (Figure 1). In 7/16 hrHPV cases (43.8%), Ki67 was limited to the basal layer (Table 1).

**Figure 2:**
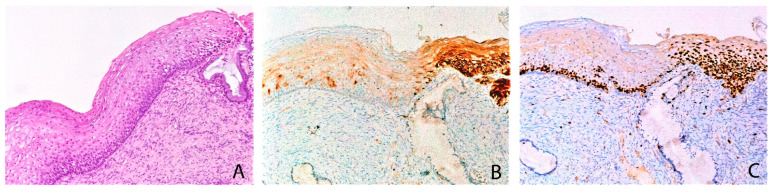
w/f p16+ CAL case with mild epithelial atypia (**A**) characterized by weak and patchy p16+ immunostaining in contiguity to a circumscribed “block like” p16 positive area (**B**), the latter with full thickness Ki67 immunostaining (**C**). A: Ematoxylin and eosin, 100×; B: p16 immunohistochemical stain, 100×; C: Ki67 immunohistochemical stain, 100×.

In some cases, in the background of w/f p16+ immunostaining, small and circumscribed “block-like” p16 positive areas (less than 50% of the epithelium) with full thickness Ki67 immunostaining were present (Figure 2).

### 3.2. p16-CALs

HPV DNA test: 19/20 p16- CALs (95%) were negative for HPV-DNA. One case (5%) showed the presence of hrHPV DNA (genotype 16).

Ki67 immunostaining: Ki67 expression was limited to the basal layer of the squamous epithelium in all cases.

The statistical analysis, performed using a Chi-square test with Yates correction, showed a significant statistical difference for the presence of hrHPV (Groups 1 and 2 of the IARC classification) between the two groups (*p* values < 0.01).

In the p16 negative surgically resected control cases, HPV DNA was absent and Ki67 immunostaining was limited to the basal layers.

Table 1 shows the results of the p16 and Ki67 immunostaining, HPV genotypes, and multiple infections in w/f p16+ CALs and in p16- CALs.

## 4. Discussion

Cervical biopsy represents a crucial point in the management of patients with cervical cancer. Cases with low-grade dysplasia and persistent hrHPV DNA detection need careful follow-up, due to the risk of progression over the time. P16 immunostaining has been considered a useful surrogate for transcriptionally active hrHPV infection, but only strong and diffuse (“block like”) positivity is considered specific. Recently, Yuxin Liu and al. [18] emphasized the existence of a wide spectrum of p16 immunostaining patterns not fulfilling LAST criteria [10]. They defined “ambiguous” patterns as consisting of (1) strong, diffuse, continuous immunostaining of the lower third of the epithelium without upward extension; (2) weak, diffuse, discontinuous immunostaining reaching at least two-thirds; and (3) strong, focal, and discontinuous immunostaining located at any level of the epithelium. They found high risk HPV DNA (including HPV 16 and 18 genotypes) in 30% of these cases.

Noteworthy, they did not investigate cases with weak, focal, and discontinuous p16 staining, which they considered as a “negative pattern”. To our knowledge, no systematic studies have been performed to assess the meaning of this w/f p16+ pattern in CALs.

In this study, hrHPV (or possible hrHPV) genotypes were found in 16/20 (80%) w/f p16+ CALs, often as the co-infection of multiple genotypes. Moreover, 9/16 (56%) cases showed a high proliferation rate, with Ki67 nuclear immunostaining in all epithelial layers, suggesting that a transcriptionally active viral phase is ongoing.

The remaining 7/16 (43.8%) w/f p16+ CALs with a low proliferative activity (Ki67 not increased, limited to the basal layers) probably represent latent, relatively harmless, HPV infection with less clinical consequences, if underdiagnosed.

Of note, 19/20 p16- CALs were negative for HPV DNA presence, suggesting that they are metaplastic and reactive lesions unrelated to HPV, in keeping with previous studies [19,20]. Only 1/20 cases showed the presence of hrHPV genotype 16, in which p16- immunostaining probably indicates a transcriptionally non active viral phase, in keeping with the low Ki67 expression.

P16 immunohistochemistry was hypothesized to distinguish between transforming and non-transforming HPV infections, based on the concept that HPV-mediated transformation is triggered by the dysregulated expression of the viral oncogenes E6 and E7 in the basal and parabasal cells.

However, although the basal and parabasal cells are considered to be the ones in which transformation begins, only the block positive p16 expression pattern was defined as a hallmark of HPV-dependent transformation, and thus considered as p16+ [10,21,22].

The results of the present study suggest that weak and focal p16 expression is not a negative or non-specific result. According to the hypothesis that transformation begins from basal and parabasal cells, it could be the early pattern of transforming HPV infection, as demonstrated by the strongly relation with the presence of rarer hrHPV genotypes and by the increase of ki67 immunoreactivity in half of the w/f p16+ cases.

In fact, the w/f p16+ pattern showed 80% sensitivity, 95% specificity, 94.12% positive predictive value (PPV), and 82.61% negative predictive value (NPV) for hrHPV infection.

The coexistence of areas with circumscribed “block-like” p16 immunoreactivity and the increase of Ki67, in a background of w/f p16+ expression, suggests that the w/f p16+ pattern could evolve in a “block-like” p16 immunoreactivity if the infection persists.

If these results are confirmed, HPV DNA testing and a follow-up should be recommended, at least for cases with increased Ki67 expression.

The study presents some limitations. The first limitation is the small number of cases, and the second is the short follow-up period. Nevertheless, this is a pilot study, and it has some strengths, as follows: the first is the high PPV and NPV for hrHPV infection. The second is the capability of highlighting the presence of rarer hrHPV genotypes at the very early stages of infection, allowing patients to be subjected to an accurate follow-up.

This is in keeping with recent studies highlighting the importance of the use of p16 immunostaining, alone or in combination with extended HPV genotyping, to improve the screening programs and the subsequent management of the patients [23,24,25].

The results of long-time follow-up data will be reported in future studies.

Remarkably, HPV16 or 18 genotypes were not detected in w/f p16+ cases, probably because HPV16 and 18 have a faster transforming power, quicky reaching the block p16+ stage, making hard to detect the w/f p16+ in the early stage. To confirm this hypothesis, further studies are necessary.

Most low-grade lesions spontaneously regress in few months, probably due to immunological intervention. Morphological criteria alone are not sufficient to distinguish lesions that may regress from those that might persist and progress.

Cases with a w/f p16+ expression may represent an early stage of transformation related to the less common hrHPV genotypes, probably with a slower transforming power and perhaps less prone to persist. Ki67 immunostaining, HPV DNA extended genotyping, and follow-up should be warranted to evaluate, in case of hrHPV positivity, the subsequent evolution (perhaps slow but potentially dangerous), into overt dysplastic changes.

The present study and future follow-up reports could highlight the benefits of a greater attention to weak/focal p16+ cases, improving the knowledge of rarer hrHPV genotypes, with a positive impact on cervical cancer screening programs.

## Figures and Tables

**Table 1:** Cases 1–20: Cervical atypical lesion with focal and weak p16 staining (w/f p16+ CALs). Cases 21–40: cervical atypical lesion negative for p16 immunostaining (p16- CALs).

Cases	p16 **		Ki67 *	High Risk HPV (IARC Groups)		Low-Risk HPV	HPV Negative
	w/f	neg		I	II		
1	x		1		53		
2	x		1	31	66		
3	x		2	51	66,73		
4	x		2	31			
5	x		2		26		
6	x		2	59			
7	x		1	51	26		
8	x		2	51	82		
9	x		2		73		
10	x		1	31	73		
11	x		1	56			
12	x		1	51	66		
13	x		2	31	73		
14	x		2	56			
15	x		2		26		
16	x		1		53		
17	x		1			54	
18	x		1				x
19	x		2				x
20	x		1				x
21		x	1				x
22		x	1				x
23		x	1				x
24		x	1				x
25		x	1				x
26		x	1				x
27		x	1				x
28		x	1				x
29		x	1				x
30		x	1				x
31		x		16			
32		x					x
33		x					x
34		x					x
35		x					x
36		x					x
37		x					x
38		x					x
39		x					x
40		x					x

* p16: w/f = weak and focal immunostaining pattern; neg = negative immunostaining pattern; ** Ki67: 1 = positive nuclear immunostaining in basal layers of squamous epithelium; 2 = positive nuclear immunostaining in all the squamous epithelium layers.
